# Optical Assessment of Porosity Parameters in Transparent Woven Fabrics

**DOI:** 10.3390/polym13030408

**Published:** 2021-01-27

**Authors:** Klara Kostajnšek, Živa Zupin, Aleš Hladnik, Krste Dimitrovski

**Affiliations:** Department of Textiles, Graphics Art and Design, Faculty for Natural Sciences and Engineering, University of Ljubljana, Snežniška 5, 1000 Ljubljana, Slovenia; ziva.zupin@ntf.uni-lj.si (Ž.Z.); ales.hladnik@ntf.uni-lj.si (A.H.); krste.dimitrovski@gmail.com (K.D.)

**Keywords:** pore size, pore size distribution, porosity parameters, weaves, woven fabrics, cover factor, image analysis

## Abstract

This paper deals with the possibility of a fast and accurate assessment of the number, size, and distribution of pores in transparent woven fabrics based on light penetration. The procedure of analyzing the pore structure in the fabrics based on a digital image is presented in detail. Fabric pores are treated as image particles and analyzed with the Java-based image processing software ImageJ. The obtained data relate to the constructional parameters of the fabric that allow for further analysis, provide the possibility to compare structurally similar or different samples as well as double check the results generated by optical or other means. This paper describes work on plain and similar to plain weaves. The conducted analysis revealed several expected and some unexpected results. Among the former, we can list the range of pore sizes in the examined woven fabrics, the distribution of pores in regard to their similarity, and the effect of dents. Examples of the latter are the magnitude of the cumulative percentage of pores in regard to the weave and the degree to which they participate in the inter-yarn and inter-fiber pores.

## 1. Introduction

Evaluation of the porosity parameters (number, size, and distribution of pores) of woven fabrics is of great importance as they strongly influence many permeability properties where they play a crucial role during their usage. The media that penetrate through the fabric may be the following: UV light, air and other gases, water and other liquids, water vapor and heat, bacteria, sound penetration, etc.

There are many types of methods for evaluating porosity parameters, such as: intrusion/extrusion methods, fluid flow methods, sieving methods, geometric and optical methods. From the number of types of methods, it can be observed that none of them give satisfactory results in all application areas. Each of them has some advantages and disadvantages. 

Mercury intrusion and liquid extrusion methods use a liquid that is being intruded to or extruded from the primary saturated sample. The size and distribution of pores are calculated based on the surface tension of the liquid and the pressure used for its intrusion or extrusion. The measured value is the volume of intruded or extruded liquid, in regard to the used pressure. The methods mainly reveal volume pore distribution in regard to their diameter. During the examination, the samples are often exposed to deformation because detection of the smallest pores requires the usage of high pressure drops [1,2].

Fluid flow methods also calculate the distribution of pores by using presaturated liquid samples. The changes in the flow are measured with respect to the opening of the pores under increasing pressure drop. The methods give a distribution of minimal diameter of the pore channels in the samples [3,4]. The fluid flow method for determining the average pore size in the samples exists. This method is based on consecutive measurement of air flow in its laminar part through dry and not saturated samples. However, in that case, the distribution of pores is unknown [5]. All methods mentioned up to now are suitable also for non-transparent fabrics, and all, except the last mentioned one, are time-consuming and need expensive equipment.

Sieving methods are used mostly in the geotextiles area. These types of methods are based on the permeability of granulated particles of known dimensions passing through the textile barrier under specific conditions. After applying mechanical action to the particles (sieve shaker method), it is possible to measure the number of particles penetrating through the fabric. Repeating this procedure several times with the differently calibrated particles indicates the distribution of pores in the sample. The methods are also convenient for non-transparent fabrics, mostly non-woven, but are also test materials and time consuming [6,7,8].

Finally, both optical and geometrical methods for determining porosity parameters in woven fabrics are discussed. The intention of the authors is to use the combination of both for obtaining added value by extending the parameters that better describe the internal geometrical structure of woven fabrics. Among other aspects, this allows for easy determination of yarn diameter in the deformed form, cumulative percentage of the area, and defining the number of pores, in regard to their diameters. This also provides an easy way of determining the characteristics of inter-yarn and inter-fiber pores. Initially, in the theoretical part, we present the advantages and disadvantages of optical methods and the geometrical method.

When discussing the disadvantages of optical methods, we can list them as follows: the methods are only suitable for transparent fabrics; they depend strongly on the quality of the images taken; they show porosity parameters as two-dimensional, therefore the thickness/length of the pores is ignored; show only the minimum transparent area of the pores. The advantages of optical methods are the following: very easy acquisition of images under different magnifications, fast and accurate image processing with already existing software, the possibility of double checking the results, and the possibility of comparing the results with other types of methods [9,10,11]. A variety of contemporary equipment for taking images, including microscopes and scanners, are available today. This equipment may be widely used in different conditions. Additionally, software for image processing exists as a part of the microscope software, independently, or as open source software, such as ImageJ. Some measurements can be calculated easily, either individually or in conjunction with novelties in the theoretical treatment of fabric geometry (Equation (11)), and in this way, parameter measurement can be double checked. When we discuss the double control of some porosity and other structural parameters, we consider the possibilities of direct measurement of the width and length of pores, measurement of distances from yarn to yarn (in this way, warp and weft densities are expressed very precisely), and measurement of the diameter of yarns (in this way, the deformed diameter of the yarn in the woven structure is determined). All the above-mentioned measurement methods are traditionally available, but they are limited by the usual and known imperfections of textile materials (deformability of the yarns, hairiness, etc.), or by requirements for a large number of measurements and their statistical processing in order to obtain satisfactory/accurate results. Based on the constructional characteristics of woven fabrics and their theoretical connection with porosity parameters, ImageJ analyses allow very fast and more accurate results, including double checking of the results.

Optical methods for assessing the woven fabrics and their surface properties are generally widely used, mostly for assessing pilling effects, wrinkling, surface color, imperfection of fabrics (online detection of fabric defects), etc. However, not many deal with porosity parameters—the number, size, and distribution of pores. The problem is presented in two parts: first, how to obtain the proper image and second, how to process and interpret the measured results for practical use.

Gong and Newton, 1992 [12] describe a method for determining pore size distribution in a fabric with images captured by a video camera using digital processing techniques. The paper presents a method for determining pore sizes, hydrodynamic diameter, its circumference and “thickness”, and the diameter of the maximum inscribed circle. The latter should lead to distributions of pore size that most closely correlate with the laboratory measurement of the pore size of a fabric which they use. Çay et al., 2005 [10] presented a paper in which an image analysis technique was used to measure the quantity of light passing through a set of 30 woven fabrics. After MATLAB processing of the measurements, the results obtained were compared with the air permeability of the fabrics and a linear relationship was found between the brightness and the percentage of air permeability. In subsequent work [13,14], an attempt was also made to predict air permeability using an artificial neural network [14]. Tàpias et al., 2010 [15] conducted a study on how to objectively measure the cover factor of woven fabrics. It precisely describes a procedure that has results which are also important for our research, since we are concerned with the opposite—the percentage of open area in woven fabrics. Angelova, 2012 [16] presented a paper with a very similar title to ours. The research is based on the development of Computational Fluid Dynamics (CFD) for numerical modeling of textile structures to predict their properties, even without having them in a physical form. For this purpose, image analysis was used to determine porosity parameters, especially of different sized and shaped areas of transparent woven fabrics. It was not possible to distinguish between inter-yarn and inter-fiber pores, which may be a reason for large discrepancies between the theoretical and measured results. Turan and Okur, 2012 [17] calculated the inter-yarn porosity, pore size, and pore size distribution of cotton woven fabrics using two-dimensional (2D) and three-dimensional (3D) geometric pore models and also used the image analysis method. After MATLAB processing, the differences between weave types with the same structural parameters show that the 3D fluid flow mechanism is a complex mechanism influenced not only by porosity or pore size, but also by 3D pore shape, which shows the disadvantage of the image analysis method. Ragab et al., 2017 [11] presented a protocol to determine the pore size and pore size distribution in a woven structure by image analysis techniques. They followed the image analysis with Hagen–Poiseuille equations in laminar flow through a non-circular and irregular pore structure. They also used a fluid flow method to determine the size and distribution of the pores. They found a very good correlation between the distribution of pores determined by image processing and the fluid flow method. The fabrics studied were transparent and light (up to 131 g/m^2^). The distribution curve was a deformed normal distribution, which was expected since there were no differences among inter-yarn and inter-fiber pores. Owczarek, 2019 [18] presented a work on the evaluation of inter-yarn pores in fabrics using a self-developed software. The software provides pore characteristics the way equivalent to the ImageJ software. The obtained measurements were supported with the measurements of air permeability and thus, a good correlation was found between the inter-yarn pores (called ITP—inter-thread pores) and air permeability, which means that the flow through the inter-fiber pores can be neglected. 

In our investigation, we will start with a properly acquired image. All image analysis measurements are primarily based on the number of pores and their area and then on the shape, perimeter, center of mass, etc. We will process the image analysis results using ImageJ (open source software, large scale validated, and free for use) and present the processing technique in detail. We will combine the results of image analysis with the structural geometric characteristics of woven fabrics. We expect to obtain a range of interpreted results much closer to textile engineers and the final user. In addition to the distribution of pores in regard to their diameter, we expect the results to find a cumulative percentage of pores and open areas of fabrics, where the number and friction of inter-yarn and inter-fiber pores can be easily detected. We expect that in this way, fabrics can be easily compared.

## 2. Theoretical Part

### 2.1. Pore Structure of Woven Fabrics

Porosity is defined as the volume of air in the total volume of the body. It can be presented with Equation (1). Since the volume of air in the body can consist of a small amount of relatively big pores or a large amount of relatively small pores, porosity itself as a physical parameter of fabrics does not present a sufficient parameter for comparing fabrics, especially their permeability properties. For that reason, it is necessary to consider the so-called porosity parameters—the number, size, and distribution of pores. Those parameters give detailed information about the internal geometrical structure of fabrics and are related with the permeability properties of the fabrics [19,20].

(1) $$\varepsilon =\frac{V_{\mathrm{air}\;\mathrm{in}\;\mathrm{fabric}}}{V_{\mathrm{total}\;\mathrm{volume}\;\mathrm{of}\;\mathrm{fabric}}}=1-\frac{\rho_{\mathrm{fabric}}}{\rho_{\mathrm{fibre}}}$$

where ρ_fabric_ and ρ_fibre_ present the physical densities of fabric and fibers, respectively.

**Figure 1:**
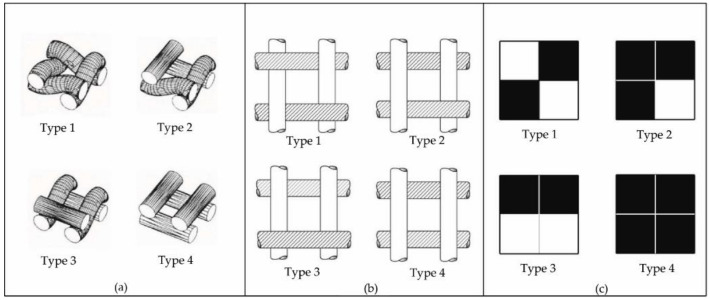
Four types of pores in woven fabrics, shown: three-dimensionally (**a**), planar (**b**), and on woven paper (**c**) [19,21].

Understanding of the optical evaluation of porosity parameters is closely related to the planar, geometric representation of one-layer woven fabric structures. There are four types of pores in one-layer woven fabrics (Figure 1). Depending on the type of weave, one-layer woven fabrics can consist of one type of pore (plain and twill weave 2/2), two types of pores (twill weave 1/2), three types of pores (twill weave 1/3), or sometimes even four types of pores. The pores have a rectangular shape and differ in dimension, texture of the pore walls, length, and positioning of their minimal diameter. The planar structure of the pores shown in Figure 1b does not take into account the third dimension of the pores, but allows the calculation of the hydraulic diameter of the pores, which transfers the two dimensions (length and width) of the rectangular pores to only one dimension (diameter) of the cylindrical shape according to Equation (2).

(2) $$d_{h}=2\times a\times b\;/\left(a+b\right)$$

where a presents the width and b presents the length of rectangular pores. 

This simplifies the presentation of the pores and makes it closer to the prediction of some permeability properties (air permeability) [21,22]. The most significant disadvantage of such defined hydraulic diameter of the pores is that, with the same constructional parameters in the woven fabric construction (fineness of the yarns and densities), the result value is always the same, regardless of the pore type in the woven structure. It is important to note that at the same time, the permeability, e.g., of air, may be significantly different.

The absence of the properties of the pores in the third dimension does not allow a direct, accurate connection between the hydraulic diameter of the pores and the air permeability. For a more accurate prediction of air permeability, another variable must be included in the equation. Apart from the number of pores on a square area, in our previous research, we used the total porosity of woven fabrics as compensation for the missing data of the third dimension. The three variables were chosen because they can easily be determined from primary constructional parameters and the physical properties of woven fabrics, i.e., theoretical diameter of the yarns, density of the yarns, and thickness and mass per square meter, from which the total porosity is calculated. The linear combination of all three variables covers a large amount of the accurate prediction of the air permeability of woven fabrics (Equations (3) and (4)).

(3) $$Q=f\left(d_{h},n,\varepsilon \right)$$

(4) $$Q=\left(k_{1\;}\times d_{h\;}\pm k_{2\;}\times n\;\pm k_{3\;}\times \varepsilon \right)$$

where d_h_ is the hydraulic diameter of the pores, n is the number of pores in the square area, ε is the total porosity of a woven fabric, and the coefficients k_1_, k_2_, and k_3_ are obtained from multiple linear regression [9].

### 2.2. Extended Woven Fabrics Cover Factor Theory

The theory of cover factor indicates the part of the area covered by yarns. In general, two cover factors are known [22,23]. One of them is the cover factor related to the one system of yarns only (warp or weft). The second is the cover factor of the fabric itself. They are described mathematically in the next three equations:

(5) $${\mathrm{Cf}}_{\mathrm{wa}}=\frac{d_{\mathrm{wa}}}{S_{\mathrm{wa}}}=\frac{d_{\mathrm{wa}}}{\left(\frac{1}{D_{\mathrm{wa}}}\right)}=d_{\mathrm{wa}}\times D_{\mathrm{wa}}$$

(6) $${\mathrm{Cf}}_{\mathrm{we}}=\frac{d_{\mathrm{we}}}{S_{\mathrm{we}}}=\frac{d_{\mathrm{we}}}{\left(\frac{1}{D_{\mathrm{we}}}\right)}=d_{\mathrm{we}}\times D_{\mathrm{we}}$$

(7) $$\mathrm{Cf}={\;\mathrm{Cf}}_{\mathrm{wa}}+{\mathrm{Cf}}_{\mathrm{we}}-{\mathrm{Cf}}_{\mathrm{wa}}\times {\mathrm{Cf}}_{\mathrm{we}}$$

where Cf_wa_ and Cf_we_ present the cover factor of warp and weft yarns, d_wa_ and d_we_ present the diameter of warp and weft yarns, S_wa_ and S_we_ distance between warp and weft yarns, and D_wa_ and D_we_ present the density of warp and weft yarns.

In general, there are three groups of woven fabrics: fabrics with the same warp and weft threads and the same densities; fabrics with the same warp and weft threads but different densities; and fabrics with different yarn fineness and different densities. For this research, only fabrics with the same construction parameters in warp and weft direction are used, so-called square fabrics. 

In the case of identical yarns and identical densities of warp and weft yarns (d_wa_ = d_we_ and D_wa_ = D_we_), Equation (7) transforms into (8) and (9) [23,24]:

(8) $$\mathrm{Cf}=2\times {\mathrm{Cf}}_{\mathrm{wa},\;\mathrm{we}}-{\mathrm{Cf}}_{\mathrm{wa},\;\mathrm{we}}^{2}$$

(9) $$\mathrm{Cf}=2\times d_{\mathrm{wa},\;\mathrm{we}}\times D_{\mathrm{wa},\;\mathrm{we}}-d_{\mathrm{wa},\;\mathrm{we}}^{2}\times {\;D}_{\mathrm{wa},\;\mathrm{we}}^{2}$$

This is a square equation with two real solutions. If we consider (1 – Cf) = Op (where Op represents open area for light), the solution is presented as Equations (10) and (11) [24]:

(10) $$d_{\mathrm{wa},\;\mathrm{we}}=\frac{1\pm \sqrt{\left(1-\mathrm{Cf}\right)}}{D_{\mathrm{wa},\;\mathrm{we}}}=\;\frac{1\pm \sqrt{O_{p}}}{D_{\mathrm{wa},\;\mathrm{we}}}$$

The physical meaning has the solution with the negative sign:

(11) $$d_{\mathrm{wa},\;\mathrm{we}}=\frac{\left(1-\sqrt{O_{p}}\right)}{D_{\mathrm{wa},\;\mathrm{we}}}$$

If we choose the appropriate density for the appropriate value of the open area of the fabric, it is possible to calculate the diameter of the thread or vice versa, which is checked during research.

### 2.3. Short Presentation of ImageJ

The free Java-based image processing and analysis software, ImageJ [25], is an open source software for image analysis. It is a highly developed software that has existed for a few decades, it is continuously improving, and supports different kinds of measurements based on the graphical elements of the presented pictures/images. It is used in all fields of science and technology [6,8,12,15,26].

The method of image analysis is based on the transmission of visible light through a material. ImageJ software supports analyzing the image, measuring distances and angles, creating histograms, and it supports standard and advanced image processing functions, etc. The software can statistically evaluate the results of measurements on individual pixels or on the area of the image specified by the user. Using spatial calibration, real physical dimensions can be assigned to pixels. The software also allows automatic use of a default threshold, displays spreadsheets, and provides diagrams. For more detailed information about the area, shape, and volume of individual parts (pores), we can use image analysis to look at the frequency distribution of the values of parameters or describe another area of particles, which gives us information about: the determination of pores, shape descriptors, perimeters, their number, size, center of mass, concentration, etc. [12,18,26,27].

A lot of other information can be obtained from the method described, such as standard deviation, the minimum and maximum grey value, the mean grey value, the modal grey value, the median, the limit to a threshold value, etc. It is also possible to determine additional geometric parameters, such as: center of gravity, equivalent circle diameter, circumference, maximum diameter, orientation of the maximum diameter, width, Feret’s diameter, number of neighboring elements, curvature, etc. [6,8,26]. The examples presented on the ImageJ website do not contain examples of usage in woven fabrics. The idea of the authors is to take the pores in transparent woven fabrics as particles, and use the ImageJ software to evaluate the porosity parameters.

## 3. Materials and Methods

### 3.1. Materials

In the experimental part, we analyze plain and similar to plain (basket, warp, and weft rib) woven fabrics made from the same yarns (fineness: 36 tex) and the same densities (24 yarns/cm) in warp and weft. The reason for this is to analyze the inter-yarn pores between the yarns, which interlace differently and are the pores of the plain type. Pores of other types (pores between yarns that interlace identically) are disregarded in order to test the theory that the pores do not participate equally in permeability properties.

### 3.2. Methods/Procedures

**Figure 2:**
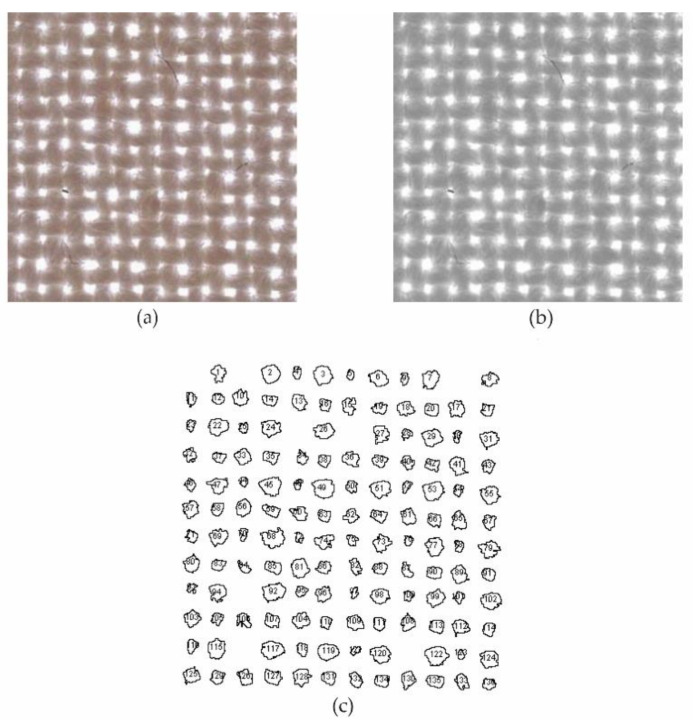
Visually displayed 2, 3, and 4 steps of image processing. (**a**) Image of plain woven fabric cropped on 5 mm × 5 mm; (**b**) the same image after binary transformation; and (**c**) different pore shapes and numbered particle size pores with an area larger than 0.01 mm^2^.

We took the pictures of the woven fabrics using a stereomicroscope (Leica Microsystems GmbH, Wetzlar, DE) under 10 X magnification and studied the acquired images with ImageJ. The processing is described in the following steps and shown in Figure 2:

1. Adjustment of the scale based on the magnification/condition of the acquired microscopic image.
2. Cropping the image to the desired/necessary dimensions.
3. Transforming the image into a binary form by thresholding it using default settings.
4. Analyzing the pores in images as particles under different initial ranges of pores from 0 to infinity. The computer generates all the measured values that were requested.
5. Repeating the procedure several times with different increasing starting points to allow additional calculations—including the average area and the average diameter of pores and their distribution.

After finishing step 5 in the listed procedure, ImageJ outputs the results of the required measurements in the form of Table 1.

The numbers in the white colored columns are the numbers that ImageJ outputs after the processing, and the numbers in the grey colored columns are the results of additional calculations, considering the basic structural parameters of the investigated fabrics.

The first column in Table 1 shows the range of the particles’ area from x to infinity in mm^2^. We started with pore area sizes from 0 to infinity; if we want particles of which sizes are larger than e.g., 0.01 mm^2^, we enter 0.01–infinity. If we want to limit the maximum value, we enter the maximum desired size instead of infinity. When determining the threshold value of the size of the examined particles/cells, it is important to make sure that no information is lost due to possible “deletion”.

The second column shows the number of particles/pores in the specified range. The following two columns show the total area of the pores and their average size in mm^2^. The last two columns present the open area fraction in % and the average perimeter of the pores in mm. By observing the first six columns of Table 1, we can conclude that in the measured area of 5 mm x 5 mm exist 189 pores with a total area of 4.9982 mm^2^, which is slightly less than 20% of the area fraction. The average value of the area is 0.0264 mm^2^ and the average perimeter of the areas is 0.6335 mm. By setting the range of pores area to 0.01 to infinity and further, we obtain a smaller number of pores, a smaller total area, and a smaller area fraction. The problem is deciding which results can be taken as representative for the presented sample. In this case, we have used known facts given by the number of inter-yarn pores. Since the density of the warp and weft was equal to 24 ends/picks per cm, we know that there are about 144 pores in an area of 5 mm x 5 mm. The result that shows the closest number of pores can therefore be considered the most realistic, and the other pores are probably pores formed by fibers. Importantly, the measurements presented allow further processing of the parameter, leading to new parameters much more familiar to the textile engineer and especially users. Some of them are shown in Table 1 and finally, in Table 2.

In the seventh column of Table 1, the diameter of the yarns is calculated according to Equation (11). It is expected that with the increase in the range of the registered area of pores, the total open area portions diminish and the diameter of the calculated yarn consequently increases. The real diameter of the yarn can be included in the calculated diameter from the real number of inter-yarn pores (239.89 µm) or, preferably, it can be calculated directly from the portion of open area in the range from 0 to infinity—in our case, 235.5245 µm.

In the eighth column of Table 1, the values of the hydraulic diameters of the pores are calculated according to Equation (2) or Equation (12):

(12) $$d_{h}=\frac{\left(4\times P\right)}{O}=\;\frac{\left(4\times A.S.\right)}{P_{p}}$$

where d_h_ represents the hydraulic diameters of the pores, P the area and the O perimeter, A. S. the average size (4th column in Table 1), and Pp perimeter (6th column in Table 1).

As the area in the ranges increases faster than the perimeter, the values of d_h_ increase from about 0.073 mm.

The ninth column of Table 1 gives the calculated hydraulic diameter of the pores (d_h1_) in every range calculated from the density of the yarns (D_wa, we_) and their diameter. For a squared construction, Equation (13) is simple:

(13) $$d_{h1}=\frac{1}{D_{\mathrm{wa},\;\mathrm{we}}{}_{\;}}-d_{\mathrm{wa},\;\mathrm{we}}$$

Due to the increasing diameter of the yarns, the calculated hydraulic diameter decreases.

The last grey column of Table 1 indicates the average diameter of the pores, which was calculated as the average size corresponding to the area of the cylindrical pore according to Equation (14):

(14) $$d_{p}=\sqrt{\frac{4\times A.S.}{\pi }}$$

Since the average size increases with the range, the average diameter of the pores also increases. However, we can assume that, as the average diameter of the pores is the one that corresponds to the actual number of pores, this is the result of the differently interlaced warp and weft yarns.

Based on the results of Table 1, we have compiled Table 2, which shows the area range (Range) in column 1, the number of pores in every range (ΔCount) in column 2, the percentage of pores in every range (ΔCount) in column 3, and the cumulative percentage of pores (C. ΔCount) in column 4. The values corresponding to 0.01 mm^2^ are taken as 100%. Columns 5, 6, and 7 are positioned in the same way, representing the area in a certain range (ΔT. A.), percentage of areas in ranges (ΔT. A.), and cumulative percentage of areas (C. ΔT. A.). As previously, the values corresponding to 0.01 mm^2^ are taken as 100%. The reason for this is to show what the measurements below this area represent: the number, percent, and cumulative percent of pores and the areas that mostly belong to inter-fiber pores. The last column shows the average diameter of the pores in every range calculated from the data in Table 2 using Equation (15):

(15) $$d_{p}=\sqrt{\frac{4\times \Delta T.A.\;\left({\mathrm{mm}}^{2}\right)}{3.14\times \Delta \mathrm{Count}\;\left(\mathrm{no}.\mathrm{of}\;\mathrm{pores}\right)}}$$

The first row of data in Table 2 shows that the number of pores under an area of 0.01 mm^2^ represents 25.17% of the total number of pores, but only 2.32% of the total area. The confirmation that the small pores are likely to be between the fibers and not between the yarns comes in the area of pores with a diameter of only 61.6 µm. The second smallest diameter of pores is 119.580 µm, which is almost twice as large as stated.

Results presented in this way give us the possibility to present data in columns in the form of graphs that confirm what was explained and give us the opportunity to compare results between samples.

**Figure 3:**
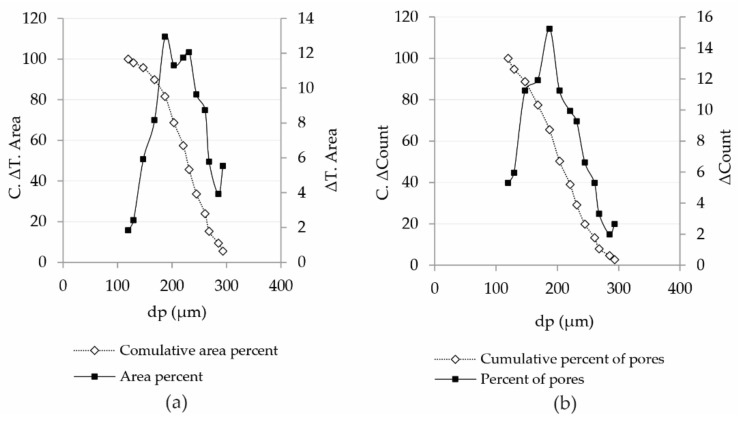
Percentage of area and cumulative percentage of area (**a**) and percentage of pores versus cumulative percentage of pores versus diameter of pores (**b**).

Table 2 (column 8) shows that the average size of pores for any consecutive range of pores increases from 119.580 to 293.453 µm. Figure 3 shows the distribution of area percentages and cumulative area percentages with respect to the diameter of pores calculated in Table 2.

Figure 3 illustrates very well the fact that there are two large groups of pores caused by denting, which is the reason why the curve does not take the normal distribution shape. This is essentially confirmed by the second diagram, where it can be observed that the percentage of the number of pores with smaller diameters does not decrease radically, which is the case with the percentage of area.

Table 2 shows that the values of the average diameter of the pores will be in the range between 119.580 and 293.453 µm. It must be noted that if we calculate the hydraulic diameter of pores, we will obtain the average diameter of pores defined from Equation (11), 173.1 and 178.49 µm, as is shown bolded in Table 1.

## 4. Results and Discussion

Table 3 shows some measured characteristics of the investigated woven fabrics.

In Table 3, it can be observed that there are very small differences between the fabrics among woven fabrics, considering their mass per unit area, thickness, and consequently, porosity. However, the air permeability of rib weaves and of the basket are 2 and about 2.5 times larger than the plain weave, respectively. Nevertheless, the air permeability correlates 99.448% with the number of pores between differently interlaced yarns, considering the obtained data, it can be concluded that this is the result of the difference in size, number, and distribution of pores, which is evident from the following tables. The tables show the measurement for basket (Table 4, and in Appendix A, Table A1) and rib (Table 5 and Table 6, and in Appendix A, Table A2 and Table A3) weaves, as shown in the description of the method used for the plain weave.

The results presented in Table 1 to Table 6 (and in Appendix A, Table A1, Table A2 and Table A3) served us with some numerically confirmed expected results, but we were presented with some unexpected results as well. Among the expected ones were the following:

With regard to yarn diameter, we have taken the results (Area Fraction) of each weave in the range 0–infinity and, using Equation 11, we have acquired the following results, i.e., yarn diameter for plain: 230.330 µm; for basket 235.525 µm; for warp rib 227.095 µm; and for weft rib 229.862 µm, which is 230.703 µm on average. The results differ by about 8.5 µm, which is about 3.7%. It should be noted that the usual CV% for cotton yarn of this fineness is over 10%. The results presented confirm that all fabrics are made from the same yarns in warp and weft and that there are no differences in deformability within the densities and weaves presented.

The smallest pores and the smallest range of distribution of pores have fabrics in the plain weave (from 119.580 to 293.453 µm). This is followed by ribbed weaves with larger pores and larger pore distribution (133.068 to 436.693 µm and 136.377 to 401.431 µm). The largest pores and the largest pore interval have basket weaves (125.683 to 468.906 µm).

**Figure 4:**
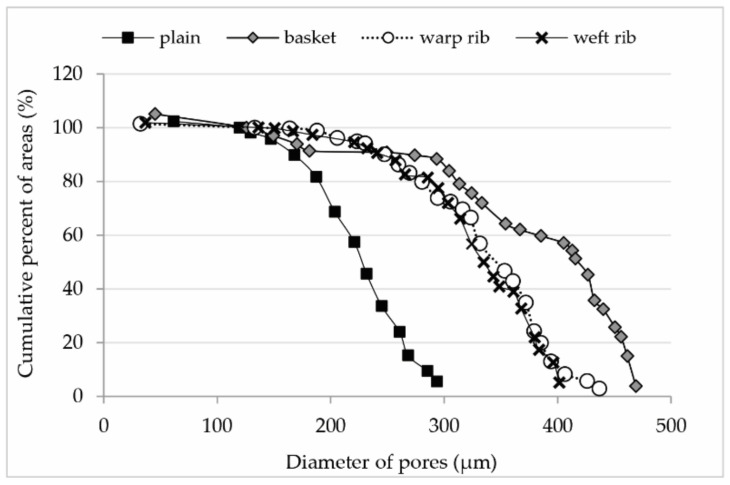
Cumulative percentage of areas in relation to pore diameter for all four weaves.

The diagram (Figure 4) shows numerically expected data. In our case, this means that almost 60% of the area in the basket weave is made of pores with a range of 400 to 470 µm, the next 30% of pores from 300 to 350 µm, and only 10% of pores from 120 to 200 µm. The same look at the rib data tells us that about 80% of the area is formed by pores of 300 to 400 µm and the other 20% by pores of 130 to 300 µm. In the case of the plain weave, 80% of the surface consists of pores of 200 to 300 µm and 20% of pores of 120 to 200 µm.

**Figure 5:**
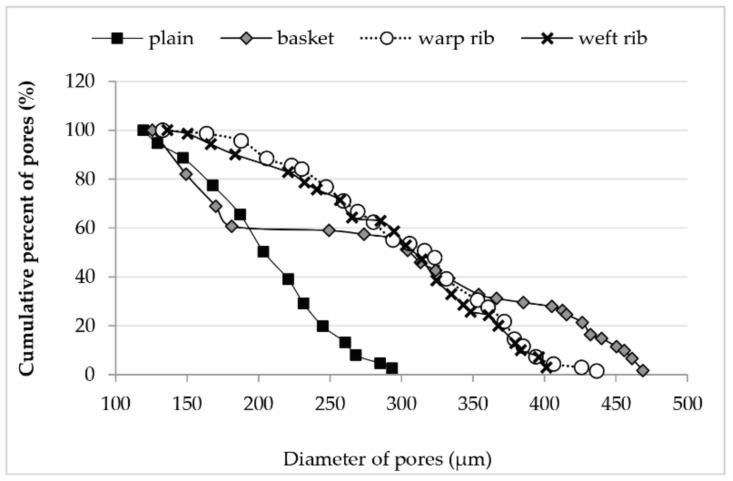
Cumulative percentage of pores in relation to the diameter of pores for all weaves.

The Figure 5 presented gives us some unexpected results. A plain weave has 40% of the pores below 200 µm and 60% between 200 and 300 µm. The pores are almost equally distributed. Rib weaves have equally distributed pores with 40% of the pores below 300 µm and 60% pores from 300 to 450 µm. The significant revelation came in basket weave where we expected two groups of pores, and in fact, there were three. About 30% of the pores are in the range of 350 to 470 µm, the next 30% are between 270 and 330 µm, and 40% of the pores are between 120 and 180 µm. The distribution of the smallest pores is almost equal with the smallest pores of the plain weave, and the distribution of pores is very similar in rib weaves in the range of 300 to 350 µm.

## 5. Conclusions

This paper discusses the possibilities of using the optical method to determine the parameters of porosity in woven fabrics: the number, size, and distribution of pores. Under the fulfilled conditions of transparent fabrics, an image with good resolution, and correct processing with ImageJ software, we can easily determine the number of pores forming areas with different ranges, both the percentage and cumulative percentage distribution. Based on these data and the data from the woven basic structure, we can decide and assess where the average diameter of the pores will be and how it fits to the conditions that the fabric should fulfill. From the known densities of yarns in woven fabrics, the number of inter-yarn pores can be easily found and the rest of the optically determined pores represet the inter-fiber pores. Furthermore, using the maximum percentage of the open area (taking all areas from 0 to infinity), we can easily calculate the diameter of yarns in the deformed form, which is one of the basic problems with textiles in general.

## Figures and Tables

**Table 1:** Presentation of ImageJ processed measurement for fabric in plain weave.

Range x–Infinity	Count (no. of Pores)	Total Area (T. A.) (mm^2^)	Average Size (A. S.) (mm^2^)	Area Fract. (%)	Perimeter (P_p_) (mm)	Diameter of Yarn (d_y_) (µm)	Hydraulic Diameter of Pores (d_h_) (mm)	Hydraulic Diameter of Pores (d_h1_) (mm)	Average Diameter of Pores (d_p_) (mm)
0	189	4.998	0.0264	20.0	0.6335	230.330	0.166693	0.186339	0.183544
0.01	151	4.885	0.0324	19.5	0.7486	232.670	0.173123	0.183995	0.203006
**0.0125**	**143**	**4.795**	**0.0335**	**19.2**	**0.7642**	**234.090**	**0.175347**	**0.182574**	**0.206681**
0.015	134	4.677	0.0349	18.7	0.7821	236.490	0.178494	0.180181	0.210866
0.02	117	4.388	0.0375	17.6	0.8123	241.870	0.184661	0.174801	0.218575
0.025	99	3.989	0.0403	16.0	0.8457	250.000	0.190611	0.166667	0.226567
0.03	76	3.357	0.0442	13.4	0.8910	264.140	0.198429	0.152525	0.237211
0.035	59	2.804	0.0475	11.2	0.9274	277.220	0.204874	0.139443	0.246070
0.04	44	2.231	0.0507	8.9	0.9597	292.360	0.211316	0.124304	0.254126
0.045	30	1.642	0.0547	6.6	0.9931	309.620	0.22032	0.107044	0.264021
0.05	20	1.171	0.0585	4.7	1.0062	326.340	0.232558	0.090331	0.273081
0.055	12	0.744	0.0620	3.0	1.0390	344.500	0.238691	0.072169	0.281073
0.06	7	0.462	0.0660	1.8	1.1012	360.7600	0.239738	0.055902	0.289959
0.065	4	0.270	0.0676	1.1	1.1310	372.9700	0.239080	0.043700	0.293453

**Table 2:** Calculation of porosity parameters based on measurements in Table 1.

Range x-Infinity	ΔCount (no. of Pores)	ΔCount (%)	C. ΔCount (%)	ΔTotal Area (ΔT. A.) (mm^2^)	ΔT. Area (ΔT. A.) (%)	C. ΔT. Area (C. ΔT.A.) (%)	d_p_ (µm)
0	38	25.166	125.166	0.1132	2.317	102.317	61.602
0.01	8	5.298	100.000	0.0898	1.838	100.000	119.580
0.0125	9	5.960	94.702	0.118	2.416	98.162	129.236
0.015	17	11.258	88.742	0.2893	5.922	95.746	147.236
0.02	18	11.921	77.483	0.3986	8.160	89.824	167.957
0.025	23	15.232	65.563	0.6323	12.944	81.664	187.138
0.03	17	11.258	50.331	0.5526	11.312	68.721	203.491
0.035	15	9.9338	39.073	0.5738	11.746	57.408	220.750
0.04	14	9.272	29.139	0.589	12.057	45.662	231.504
0.045	10	6.623	19.868	0.4708	9.638	33.605	244.897
0.05	8	5.298	13.245	0.4266	8.733	23.967	260.634
0.055	5	3.311	7.947	0.2822	5.777	15.234	268.138
0.06	3	1.987	4.636	0.1916	3.922	9.4575	285.235
0.065	4	2.649	2.649	0.2704	5.535	5.5353	293.453

**Table 3:** Physical characteristics of optically examined fabrics and air permeability at 100 Pa.

Samples/Weave	Set Number of Pores	Plain Pores among Differently Interlaced Yarns	Mass per Unit Area (g/m^2^)	Thickness (mm)	Porosity (%)	Air Permeability (cm^3^/cm^2^/s)
Plain	576	576	165.8	0.333	63.0	21.77
Basket		144	161.3	0.364	69.0	51.51
Warp rib		292	162.9	0.364	69.1	44.14
Weft rib		292	160.4	0.363	68.8	42.93

**Table 4:** ImageJ processed measurements and basic calculation for basket weave.

Range x–Infinity	Count (no. of Pores)	Total Area (T. A.) (mm^2^)	Average Size (A. S.) (mm^2^)	Area Fract. (%)	Perimeter (P_p_) (mm)	Diameter of Yarn (d_y_) (µm)	Hydraulic Diameter of Pores (d_h_) (mm)	Hydraulic Diameter of Pores (d_h1_) (mm)	Average Diameter of Pores (d_p_) (mm)
0	204	4.7323	0.0232	18.9	0.4568	235.525	0.203152	0.181142	0.171904
0.01	61	4.5041	0.0738	18.0	1.1814	239.890	0.249873	0.176777	0.306693
0.015	50	4.3677	0.0874	17.5	1.3082	242.363	0.267237	0.174304	0.333585
0.02	42	4.2276	0.1007	16.9	1.4105	245.377	0.285572	0.17129	0.358086
**0.025**	**37**	**4.114**	**0.1112**	**16.5**	**1.4875**	**247.416**	**0.299025**	**0.169251**	**0.376354**
**0.03**	**36**	**4.0882**	**0.1136**	**16.4**	**1.5043**	**247.930**	**0.302067**	**0.168737**	**0.380347**
0.05	35	4.0394	0.1154	16.2	1.5198	248.962	0.303724	0.167705	0.383433
0.055	34	3.9805	0.1171	15.9	1.5322	250.522	0.305704	0.166145	0.386184
0.065	31	3.778	0.1219	15.1	1.5725	254.755	0.310079	0.161911	0.394017
0.07	28	3.5598	0.1271	14.2	1.6077	259.655	0.316228	0.157012	0.402438
0.075	26	3.4057	0.131	13.6	1.643	263.008	0.318929	0.153659	0.40849
0.08	24	3.2409	0.135	13	1.6751	266.435	0.322369	0.150231	0.414756
0.085	20	2.8924	0.1446	11.6	1.7498	274.755	0.330552	0.141912	0.429219
0.095	19	2.794	0.1471	11.2	1.7715	277.223	0.332148	0.139443	0.432814
0.105	18	2.6885	0.1494	10.8	1.7959	279.736	0.332758	0.136931	0.436198
0.115	17	2.572	0.1513	10.3	1.8074	282.943	0.334846	0.133723	0.439012
0.125	16	2.4431	0.1527	9.8	1.8156	286.229	0.336418	0.130437	0.441038
0.13	15	2.3094	0.154	9.2	1.8172	290.285	0.338983	0.126381	0.442863
0.135	13	2.0384	0.1568	8.2	1.8359	297.352	0.341631	0.119315	0.446929
0.14	10	1.61	0.161	6.4	1.8716	311.257	0.344091	0.105409	0.452875
0.145	9	1.4633	0.1626	5.9	1.8837	315.459	0.345278	0.101208	0.455104
0.15	7	1.1593	0.1656	4.6	1.8859	327.302	0.351238	0.089365	0.459319
0.155	6	1.0000	0.1667	4.0	1.908	333.333	0.349476	0.083333	0.460776
0.16	4	0.6738	0.1685	2.7	1.8925	348.201	0.356143	0.068465	0.463234
0.165	1	0.1726	0.1726	0.7	1.9644	381.806	0.351456	0.034861	0.468906

**Table 5:** ImageJ processed measurements and basic calculation for warp rib weave.

Range x-Infinity	Count (no. of Pores)	Total Area (T. A.) (mm^2^)	Average Size (A. S.) (mm^2^)	Area fract. (%)	Perimeter (P_p_) (mm)	Diameter of yarn (d_y_) (µm)	Hydraulic Diameter of Pores (d_h_) (mm)	Hydraulic Diameter of Pores (d_h1_) (mm)	Average Diameter of Pores (d_p_) (mm)
0	157	5.179	0.0330	20.7	0.6318	227.095	0.208927	0.189572	0.204997
**0.01**	**69**	**5.107**	**0.0740**	**20.4**	**1.3104**	**228.474**	**0.225885**	**0.188193**	**0.307072**
**0.015**	**68**	**5.094**	**0.7490**	**20.4**	**1.3180**	**228.474**	**2.273141**	**0.188193**	**0.308901**
0.025	66	5.051	0.0765	20.2	1.3317	229.398	0.229781	0.187268	0.312248
0.03	61	4.913	0.0805	19.7	1.3577	231.731	0.237166	0.184936	0.320309
0.035	59	4.846	0.0821	19.4	1.3686	233.144	0.239953	0.183523	0.323481
0.04	58	4.807	0.0829	19.2	1.3670	234.093	0.242575	0.182574	0.324939
0.045	53	4.599	0.0868	18.4	1.4050	237.937	0.247117	0.17873	0.332479
0.05	49	4.407	0.0899	17.6	1.4253	241.865	0.252298	0.174801	0.338488
0.055	46	4.249	0.0924	17	1.4441	244.871	0.255938	0.171796	0.343008
0.06	43	4.078	0.0948	16.3	1.4689	248.445	0.251159	0.168222	0.347559
0.065	38	3.769	0.0992	15.1	1.5098	254.755	0.258468	0.161911	0.355452
0.07	37	3.701	0.1000	14.8	1.5352	256.372	0.260552	0.160295	0.356964
0.075	35	3.554	0.1016	14.2	1.5526	259.655	0.261754	0.157012	0.359673
0.08	33	3.397	0.1029	13.6	1.6042	263.008	0.256576	0.153659	0.362139
0.085	27	2.905	0.1076	11.6	1.6847	274.755	0.255476	0.141912	0.370211
0.095	21	2.387	0.1137	9.5	1.6847	288.241	0.269959	0.128425	0.380556
0.1	19	2.192	0.1153	8.8	1.7098	293.063	0.269739	0.123603	0.383318
0.105	15	1.783	0.1189	7.1	1.7753	305.642	0.267898	0.111024	0.389163
0.11	10	1.240	0.1240	5.0	1.9005	323.497	0.260984	0.093169	0.397508
0.115	8	1.015	0.1268	4.1	2.0139	332.298	0.25185	0.084369	0.401986
0.12	5	0.665	0.1331	2.7	2.2763	348.201	0.233888	0.068465	0.411739
0.125	3	0.422	0.1406	1.7	2.2223	362.340	0.253071	0.054327	0.423162
0.13	2	0.292	1.4600	1.2	2.5381	371.023	2.300934	0.045644	0.431336
0.145	1	0.150	0.1497	0.6	2.4712	384.392	0.242311	0.032275	0.436693

**Table 6:** ImageJ processed measurements and basic calculation for weft rib weave.

Range x-Infinity	Count (no. of Pores)	Total Area (T. A.) (mm^2^)	Average Size (A. S.) (mm^2^)	Area Fract. (%)	Perimeter (P_p_) (mm)	Diameter of Yarn (d_y_) (µm)	Hydraulic Diameter of Pores (d_h_) (mm)	Hydraulic Diameter of Pores (d_h1_) (mm)	Average Diameter of Pores (d_p_) (mm)
0	157	5.019	0.032	20.1	0.604	229.862	0.211956	0.186804	0.201795
**0.01**	**70**	**4.927**	**0.0704**	**19.7**	**1.221**	**231.731**	**0.230650**	**0.184936**	**0.299441**
**0.015**	**69**	**4.913**	**0.7120**	**19.7**	**1.229**	**231.731**	**2.316766**	**0.184936**	**0.301156**
0.02	66	4.859	0.0736	19.4	1.250	233.144	0.235482	0.183523	0.30625
0.025	63	4.794	0.0761	19.2	1.272	234.093	0.239289	0.182574	0.31134
0.03	58	4.661	0.0804	18.6	1.301	236.968	0.24729	0.179699	0.319963
0.04	55	4.547	0.0827	18.2	1.323	238.911	0.249981	0.177756	0.324506
0.045	53	4.462	0.0842	17.8	1.335	240.875	0.252379	0.175792	0.327482
0.05	50	4.325	0.8650	17.3	1.345	243.361	2.571917	0.173305	0.331962
0.55	45	4.066	0.0904	16.3	1.379	248.445	0.262295	0.168222	0.339272
0.06	44	4.011	0.0912	16.0	1.387	250.000	0.263071	0.166667	0.340764
0.065	41	3.819	0.0931	15.3	1.398	253.687	0.266476	0.16298	0.344467
0.075	37	3.546	0.0958	14.2	1.422	259.655	0.269404	0.157012	0.349414
0.08	33	3.257	0.0987	13.0	1.456	266.435	0.271154	0.150231	0.354604
0.085	27	2.793	1.0340	11.2	1.474	277.223	2.805209	0.139443	0.362991
0.09	23	2.462	0.1071	9.8	1.508	286.229	0.284066	0.130437	0.369301
0.095	20	2.198	0.1099	8.8	1.519	293.063	0.289458	0.123603	0.3742
0.1	18	2.013	0.1119	8.1	1.528	298.081	0.292932	0.118585	0.377471
0.105	17	1.918	0.1128	7.7	1.535	301.046	0.293884	0.11562	0.37911
0.11	14	1.611	0.1151	6.4	1.545	311.257	0.298013	0.105409	0.382868
0.115	9	1.080	0.1200	4.3	1.583	330.265	0.303183	0.086402	0.390945
0.12	7	0.854	0.1219	3.4	1.614	339.837	0.302107	0.07683	0.39411
0.125	5	0.623	0.1246	2.5	1.617	350.786	0.308149	0.065881	0.398277
0.13	2	0.253	0.1265	1.0	1.671	375.000	0.302831	0.041667	0.401431

## Data Availability

The data presented in this study are available on request from the corresponding author.

## Appendix A

**Table A1:** Calculation of porosity parameters based on measurements in Table 4.

Range x–Infinity	ΔCount (no. of Pores)	ΔCount (%)	C. ΔCount (%)	ΔTotal Area (ΔT. A.) (mm^2^)	ΔT. Area (ΔT. A.) (%)	C. ΔT. Area (C. ΔT. A.) (%)	d_p_ (µm)
0	143	234.426	334.426	0.2282	5.066	3.257	45.087
0.01	11	18.033	100.000	0.1364	3.028	100.000	125.683
0.015	8	13.115	81.967	0.1401	3.110	96.972	149.362
0.02	5	8.197	68.852	0.1136	2.522	93.861	170.126
**0.025**	**1**	**1.639**	**60.656**	**0.0258**	**0.573**	**91.339**	**181.291**
**0.03**	**1**	**1.639**	**59.016**	**0.0488**	**1.083**	**90.766**	**249.330**
0.05	1	1.639	57.377	0.0589	1.308	89.683	273.919
0.055	3	4.918	55.738	0.2025	4.496	88.375	293.236
0.065	3	4.918	50.820	0.2182	4.844	83.879	304.391
0.07	2	3.279	45.902	0.1541	3.421	79.035	313.294
0.075	2	3.279	42.623	0.1648	3.659	75.613	323.988
0.08	4	6.557	39.344	0.3485	7.737	71.954	333.148
0.085	1	1.639	32.787	0.0984	2.185	64.217	354.049
0.095	1	1.639	31.148	0.1055	2.342	62.032	366.599
0.105	1	1.639	29.508	0.1165	2.587	59.690	385.237
0.115	1	1.639	27.869	0.1289	2.862	57.104	405.221
0.125	1	1.639	26.230	0.1337	2.968	54.242	412.697
0.13	2	3.279	24.590	0.271	6.017	51.273	415.465
0.135	3	4.918	21.311	0.4284	9.511	45.257	426.510
0.14	1	1.639	16.393	0.1467	3.257	35.745	432.295
0.145	2	3.279	14.754	0.304	6.749	32.488	440.035
0.15	1	1.639	11.475	0.1593	3.537	25.739	450.478
0.155	2	3.279	9.836	0.3262	7.242	22.202	455.819
0.16	3	4.918	6.557	0.5012	11.128	14.960	461.328
0.165	1	1.639	1.639	0.1726	3.832	3.832	468.906

**Table A2:** Calculation of porosity parameters based on measurements in Table 5.

Range x–Infinity	ΔCount (no. of Pores)	ΔCount (%)	C. ΔCount (%)	ΔTotal Area (ΔT. A.) (mm^2^)	ΔT. Area (ΔT. A.) (%)	C. ΔT. Area (C. ΔT. A.) (%)	d_p_ (µm)
0	88	56.051	156.051	0.0718	1.406	101.406	32.239
0.01	1	1.449	100.000	0.0139	0.272	100.000	133.068
0.015	2	2.899	98.551	0.0421	0.824	99.728	163.754
0.025	5	7.246	95.652	0.1385	2.712	98.904	187.847
0.03	2	2.899	88.406	0.0665	1.302	96.192	205.807
0.035	1	1.449	85.507	0.0391	0.766	94.890	223.179
0.04	5	7.246	84.058	0.2082	4.076	94.124	230.314
0.045	4	5.797	76.812	0.192	3.759	90.048	247.278
0.05	3	4.348	71.014	0.1586	3.105	86.289	259.511
0.055	3	4.348	66.667	0.171	3.348	83.183	269.465
0.06	5	7.246	62.319	0.3086	6.042	79.835	280.400
0.065	1	1.449	55.072	0.0679	1.329	73.793	294.103
0.07	2	2.899	53.623	0.1467	2.872	72.463	305.679
0.075	2	2.899	50.725	0.157	3.074	69.591	316.228
0.08	6	8.696	47.826	0.4924	9.641	66.517	323.332
0.085	6	8.696	39.130	0.5175	10.132	56.876	331.470
0.095	2	2.899	30.435	0.1959	3.836	46.744	353.238
0.1	4	5.797	27.536	0.4082	7.992	42.908	360.555
0.105	5	7.246	21.739	0.5429	10.630	34.916	371.912
0.11	2	2.899	14.493	0.2256	4.417	24.286	379.070
0.115	3	4.348	11.594	0.3494	6.841	19.869	385.182
0.12	2	2.899	7.246	0.2437	4.772	13.028	393.983
0.125	1	1.449	4.348	0.1296	2.537	8.257	406.320
0.13	1	1.449	2.899	0.1424	2.788	5.719	425.912
0.145	1	1.449	1.449	0.1497	2.931	2.931	436.693

**Table A3:** Calculation of porosity parameters based on measurements in Table 6.

Range x–Infinity	ΔCount (no. of Pores)	ΔCount (%)	C. ΔCount (%)	ΔTotal Area (ΔT. A.) (mm^2^)	ΔT. Area (ΔT.A.) (%)	C. ΔT. Area (C. ΔT. A.) (%)	d_p_ (µm)
0	87	55.414	155.414	0.0916	1.859	101.859	36.623
0.01	1	1.429	100.000	0.0146	0.296	100.000	136.377
0.015	3	4.286	98.571	0.0533	1.082	99.704	150.442
0.02	3	4.286	94.286	0.0654	1.327	98.622	166.645
0.025	5	7.143	90.000	0.1326	2.691	97.295	183.803
0.03	3	4.286	82.857	0.1147	2.328	94.603	220.692
0.04	2	2.857	78.571	0.0846	1.717	92.275	232.132
0.045	3	4.286	75.714	0.1366	2.772	90.558	240.841
0.05	5	7.143	71.429	0.2592	5.261	87.786	256.979
0.55	1	1.429	64.286	0.0553	1.122	82.525	265.416
0.06	3	4.286	62.857	0.1918	3.893	81.403	285.384
0.065	4	5.714	58.571	0.2729	5.539	77.510	294.806
0.075	4	5.714	52.857	0.2887	5.859	71.971	303.221
0.08	6	8.571	47.143	0.4647	9.432	66.112	314.106
0.085	4	5.714	38.571	0.3303	6.704	56.680	324.332
0.09	3	4.286	32.857	0.264	5.358	49.977	334.816
0.095	2	2.857	28.571	0.1851	3.757	44.619	343.363
0.1	1	1.429	25.714	0.0953	1.934	40.862	348.427
0.105	3	4.286	24.286	0.307	6.231	38.928	361.055
0.11	5	7.143	20.000	0.5312	10.781	32.697	367.883
0.115	2	2.857	12.857	0.2263	4.593	21.916	379.658
0.12	2	2.857	10.000	0.2309	4.686	17.323	383.497
0.125	3	4.286	7.143	0.3696	7.501	12.636	396.160
0.13	2	2.857	2.857	0.253	5.135	5.135	401.431
